# Emotions and feelings in critical and emergency caring situations: a qualitative study

**DOI:** 10.1186/s12912-020-00438-6

**Published:** 2020-07-01

**Authors:** María F. Jiménez-Herrera, Mireia Llauradó-Serra, Sagrario Acebedo-Urdiales, Leticia Bazo-Hernández, Isabel Font-Jiménez, Christer Axelsson

**Affiliations:** 1 Nursing Department, Universitat Rovira i Virgili (URV), Av/ Catalunya, 35 43002 Tarragona, Spain; 2 Faculty of Medicine and Health science, Nursing Department, University Internacional of Catalonia (UIC), Barcelona, Spain; 3 Prehospital and Emergency Care, Faculty of Caring Science, Work life and Social Welfare,The Center of Prehospital Research, University of Borås, Borås, Sweden

**Keywords:** Moral emotions, Emergency care, Critical care

## Abstract

**Background:**

Moral emotions are a key element of our human morals. Emotions play an important role in the caring process. Decision-making and assessment in emergency situations are complex and they frequently result in different emotions and feelings among health-care professionals.

**Methods:**

The study had qualitative deductive design based on content analysis. Individual interviews and focus groups were conducted with sixteen participants.

**Results:**

The emerging category “emotions and feelings in caring” has been analysed according to Haidt, considering that moral emotions include the subcategories of “Condemning emotions”, “Self-conscious emotions”, “Suffering emotions” and “Praising emotions”. Within these subcategories, we found that the feelings that nurses experienced when ethical conflicts arose in emergency situations were related to caring and decisions associated with it, even when they had experienced situations in which they believed they could have helped the patient differently, but the conditions at the time did not permit it and they felt that the ethical conflicts in clinical practice created a large degree of anxiety and moral stress. The nurses felt that caring, as seen from a nursing perspective, has a sensitive dimension that goes beyond the patient’s own healing and, when this dimension is in conflict with the environment, it has a dehumanising effect. Positive feelings and satisfaction are created when nurses feel that care has met its objectives and that there has been an appropriate response to the needs.

**Conclusions:**

Moral emotions can help nurses to recognise situations that allow them to promote changes in the care of patients in extreme situations. They can also be the starting point for personal and professional growth and an evolution towards person-centred care.

## Background

Nurses in the current health-care environment are confronted by complex situations arising from the conflicting values and beliefs of other health-care professionals. In these circumstances, moral emotions arise from different feelings related to not being able to ensure the best interests of the patient and relatives. Understanding why and how moral emotions arise may help nurses to develop the caring process and make it visible to all health-care professionals. Our theory is that, if nurses are aware of their moral emotions, this will help them to cope in different situations and improve nursing practice.

### Definition of emotion

Emotions play an important role in the caring process, but there is still a shortage of articles relating nursing to emotions. Learning more about emotions is a key component in the nursing profession. The concept of emotion has multiple definitions. The lack of a definition is a constant source of numerous misunderstandings and a series of mostly fruitless debates between different disciplines [1]. In this report, we use the definition formulated by Scherer, Schorr and Johnstone in which they define emotions as an episode of interrelated, synchronised changes in all or some of the five organismic subsystems when responding to an external or internal event of concern. These five components are the cognitive system (what you think), the subjective process (how you interpret), the action tendencies (e.g. running away), the physiological changes (e.g. changes in blood pressure or size of pupil) and the motor expression (e.g. body language) [2, 3].

### How do emotions arise?

To give a brief interpretation, emotions arise from the body’s responses to external or internal stimuli. The response is dependent on your life experience, e.g. cultural factors, upbringing, education and so on [4, 5]. Feelings are a part or an expression of/from these stimuli. A feeling can trigger an emotion or be the response to one. This means that the terms “emotion” and “feelings” are used to illustrate separate actions. Emotions and feelings are often used interchangeably in everyday language.

### Moral emotions

Moral emotions, instincts, and intuitions form the moral brain, which allows people to make ethical decisions, according to Haidt [6]. These emotions are the catalyst for promoting positive actions and avoiding negative ones [7, 8]. People carry out actions and behaviours that are built on the information they obtain from previous experiences, both positive and negative. Moral emotions are the response to situations, sometimes of well-being, and sometimes of anguish or suffering of people [8]. The author classifies moral emotions into four families: condemning emotions, self-conscious emotions, praising emotions, and suffering emotions [9].

The difference between moral emotions and basic emotions is that the basic emotions come from ideas, the imagination or the perception of immediate self-realisation such as sadness, happiness, anger, disgust or joy [10]. The moral emotions are linked to the interests and/or the well-being of all people, as well as individuals. Furthermore, the moral emotions are evoked in circumstances that extend beyond the immediate sphere of self, such as empathy and compassion and, finally, the emotions relating to praising others, such as gratitude.

Finally, the main contribution to the caring ethic practices [11–14] is that it enriches our understanding of moral reasoning and decision-making. However, caring ethic practices include topics that have been ignored in rational ethical theories, such as the moral emotions.

### Moral emotions in nursing care

Nursing care is an interpersonal experience and those providing care witness emotional signals that can be described as physical, psychological or existential [15]. These signals are considered to be a moral experience to perform moral work [16]. However, the motivation to act for another individual may involve an element of personal gain and it is plausible that nurses find caring for others emotionally rewarding. A study of 56 nurses found that nurses had more empathy than other health-care professionals. The author suggested that moral emotions and empathy may be a natural part of the profession, important for nursing roles and the caring process [17]. In nurses’ experiences of care, they also found experiences of emotional guilt, anger and frustration in relation to moral conflicts. Many of these situations were patient related and associated with acts of physical care that cross physical, social and personal boundaries [18].

### Visible emotions in care situations

The interaction between the nursing professionals and other participants in the process of care is understood as an exchange of emotions, actions and experiences. In acute situations, it is necessary to focus and act quickly to continue the caring process. The arousal of feelings is secondary to the situation. It is impossible to avoid feelings, because feelings are a mental experience of body states, which arise as the brain interprets emotions.

Regardless of why emotions occur, whether or not they are appropriate or respond to certain cognitive patterns, our goal is to approach the emotions of professionals in acute care practice, emotions that arise from the interaction between the nursing professionals and other participants in the process of care. Our theory is that, if nurses are aware of their emotions, this will help them to cope in different situations. If a nurse learns to act intelligently as a result of emotions, this will improve nursing practice [19].

The overall aim of this study is to make nurses aware of moral emotions that could arise during their everyday work while taking care of patients and relatives in emergency situations.

### Aim

To analyse how emergency nurses describe the moral emotions arising from emergency care situations.

## Method

### Organisation

The study took place in Catalonia, Spain, at a university hospital and on the advanced life support (ALS) ambulance in the same town that has 131,255 inhabitants. In the present study, the aim was to select a group of nurses with experience in ALS ambulance care and emergency department (ED) care.

### Sample

The sample of participants in the study corresponds to that presented in the first part of the study where the category ethical issues was analysed [20].

Sixteen nurses aged 27–47 years agreed to participate in the study. The nurses worked at the ED, at the ALS or both units. The mean time worked was 16.86 years.

The description of the socio-demographic characteristics as well as years of experience and type of participation in the study are reflected in Table 1. All the nurses participating in the study were invited to participate in interviews and in the FG; 14 nurses took part in the interviews and 12 in the FG.

**Table 1:** Characteristics of informants

AGE RANGE	CODE	YEAR OF EXPERIENCE	FIELD	PARTICIPATION	PARTICIPATION
25–30	ENF12	6	AMBULANCE/ED	INTERVIEW	***
	ENF16	7	AMBULANCE/ED	***	FG
31–35	ENF1	8	AMBULANCE/ED	INTERVIEW	***
	ENF2	14	AMBULANCE/ED	INTERVIEW	FG
	ENF8	16	AMBULANCE/ED	INTERVIEW	***
	ENF10	17	AMBULANCE/ED	INTERVIEW	FG
	ENF13	12	ED	INTERVIEW	***
36–40	ENF5	16	AMBULANCE/ED	INTERVIEW	FG
	ENF6	15	AMBULANCE/ED	INTERVIEW	FG
	ENF15	15	AMBULANCE	***	FG
41–45	ENF3	20	ED	INTERVIEW	FG
	ENF7	22	ED	INTERVIEW	FG
	ENF9	21	ED	INTERVIEW	FG
	ENF11	23	ED	INTERVIEW	FG
	ENF14	19	ED	INTERVIEW	FG
46–50	ENF4	22	ED	INTERVIEW	FG

*** non participate at he Interview or FG

### Data collection: interview

Data were gathered using interviews. The role as interviewer was that of an encouraging, non-normative neutral facilitator so that the participants could explain themselves as fully as possible [21]. Each interview took around 90 min, was recorded on an audio file and transcribed verbatim. Transcriptions have been made after each interview to provide a clear recollection of the interview; to increase the reliability, parts of the interviews have been listened to many times. To avoid interference during data collection, this was done outside the care units.

A semi-structured interview guide was created by the authors (Table 2) to facilitate these interviews with specifics topics on the relevant experiences of the participants. In order to stimulate reflections on the research phenomenon, follow-up questions were posed such as: Could you describe the situation? Do you remember the situation in a positive or negative way? How is the atmosphere in the service? Do you have any strategies for managing your feelings?

**Table 2:** Guide notes for the interview

Guide notes for the interview	
Guide questions	Exploratory goals
Since when are you a doctor/nurse?	Age, gender, formation, years of professional experience, work fields.
Where did you study nursing/medicine?	
In which fields have you worked?	
Since when are you in the emergency department?	
What has meant for you to be a nurse/doctor?	Developed activities
What does it mean for you to take care? And to cure?	How understands the illness/ healing and caring processes
	How are perceived the persons demanding assistance
	Types of relationships built between the professionals and with who demands the services
	Satisfaction/Dissatisfaction of people
Description of situations they have lived and remember in a positive or negative way	Description of the context and situations
What does taking care feels like?	Feelings of different nature in front of lived situations
Would you change anything?	Value/exchange value
Do you have any strategies for managing your feelings?	Ethics Conflicts
	Power relations
Do patients take part of the decisions?	Caring strategies
What professionals take part when taking ethic decisions?	Communication
How is the atmosphere in the service?	Work conditions
	Relationships with the other members
	Conflicts
	Personal and professional satisfaction/ Moral distress
	Team work
	Status/Role
	Rules, strategies, tactics

### Data collection: focus group (FG)

The FG Each took around 120 min, was recorded on an audio file and transcribed verbatim. For the development, a FG guide was created (Table 3) according to help the expert in group dynamics, with some open questions from different themes arising during the interviews. In order to stimulate reflections on the research phenomenon, follow-up questions were posed such as: How is the care organised at the emergency/ED service? What kind of feelings and emotions do you have in emergency situations? How do the professionals react when faced by situations involving suffering and pain?

**Table 3:** Guide notes for the direction and development of the Focal Group

Guide notes for the direction and development of the Focal Group	
**1**	**Informed consent and autonomy:**
	Who takes the decisions about people’s health in the emergency service?
	Can the person take decisions in the emergency service?
	Whenever not accepted what is offered, what happens?
**2**	**Pain and suffering:** Is there pain and suffering in the emergency service?
	How are they dealt with?
	How does the professional react in front of these situations?
**3**	**Power relations, moral distress, people’s care management in the service.**
	How is it managed the caring in the emergency service?
	How the professional does connects with the patients in emergency situations?
	Feelings and emotions around caring in emergency situations
	How is the activity managed in the emergency service?
	And in emergencies outside the hospital, is there any difference?
**4**	**Abuse of therapeutic effort and CPR (cardiopulmonary resuscitation)**
	In extreme and terminal situations, until when is prolonged the assistance?
	Up to which point is the technique invading the human being? What is the role played by the family members?
**5**	**Information:**
	People demand more information. What happens in matter of urgency situations?
	And in emergencies? Enough information.
**6**	**Death, advance directives**
	How is dying in casualty? And in emergency services? Professional’s suffering-acceptance.
**7**	**Casualty service.** Professional’s perception of:
	Why do people come to casualty?
	Going to casualty as an alternative of treatment, why? When?...

The FG technique allowed us to deepen in aspects related to their emotions and feelings in very diverse situations and that could be contrasted among the participants. The members of the focus group share experiences with one another, they are able to highlight individual viewpoints, empower the participants and validate their experiences and be regarded as an expert [22, 23].

### Data analysis

A qualitative approach was chosen and the collected data were analysed deductively, according to content analysis [24]. The primary aim of this is to describe the phenomenon in a conceptual form from different levels of content: themes and main ideas of the text as primary content and context information as latent content. In the process of analysis, three basic forms are used: summarisation, explication and structuring.

We carried out the analysis of the material from focus groups and interviews in several steps. After the verbatim transcription of the interviews, all personal identifiers were removed or replaced and a letter and a number were attributed to each participant. Deductive category application works with previously formulated, theoretically derived aspects of analysis, connecting them with the text.

The analysis explored the data to identify patterns in the way nursing expresses the emotions based on the classification by Haidt [9] to report the experiences and the reality of the participants based on a data-driven and systematic procedure which permits searching across data sets to identify repeated patterns of meaning [25].

Within this framework, systematic stages were followed and simultaneous analysis was undertaken. (a) The transcriptions were read and the data were re-read several times to obtain a sense of the overall data; (b) the text was divided into meaning units; (c) in the abstraction process, the meaning units were coded and the codes were compared, contrasted and sorted into preliminary subcategories; (d) by going back and forth among the preliminary subcategories, the codes and the text subcategories were identified; (e) the final step in the analysis was to use the categories according with Haidt’s moral emotions families which describes the entire results and connects all the subcategories. The analysis was carried out by the main author (M.J.) and the analysis was evaluated by means of discussions between all the authors during the analysis process and by emphasizing the emotions underlying the care experiences.

### Ethical considerations

Clinic and ambulance managers were informed about the study, which they subsequently approved. This study was explained to the nurses in a group and they were told that (a) participation was voluntary and (b) they could leave the study at any time. Each individual gave her written informed consent to participate in the study.

The nurses participated on a voluntary basis and were reassured of data confidentiality. All the participants were verbal informed of the voluntary nature of the research and were told that their participation (or non-participation) would not affect their health services and after they provided written consent format to participate prior to data collection.

To maximise confidentiality, no names or other identifiers were recorded in the audio file or on the interview transcripts. The interviewers introduced the study in person and asked the participants whether they had any questions. The importance of maintaining the confidentiality of other participants, by not sharing their views outside the focus group setting, was stressed at the start of the interview.

Data from the transcripts of interviews and focus groups were collected according to the Law 15/1999 on the Protection of Personal Data. The research project was piloted and approved by the clinical committee at the reference hospital, according to Spanish law for non-biomedical studies.

## Results

**Fig. 1:**
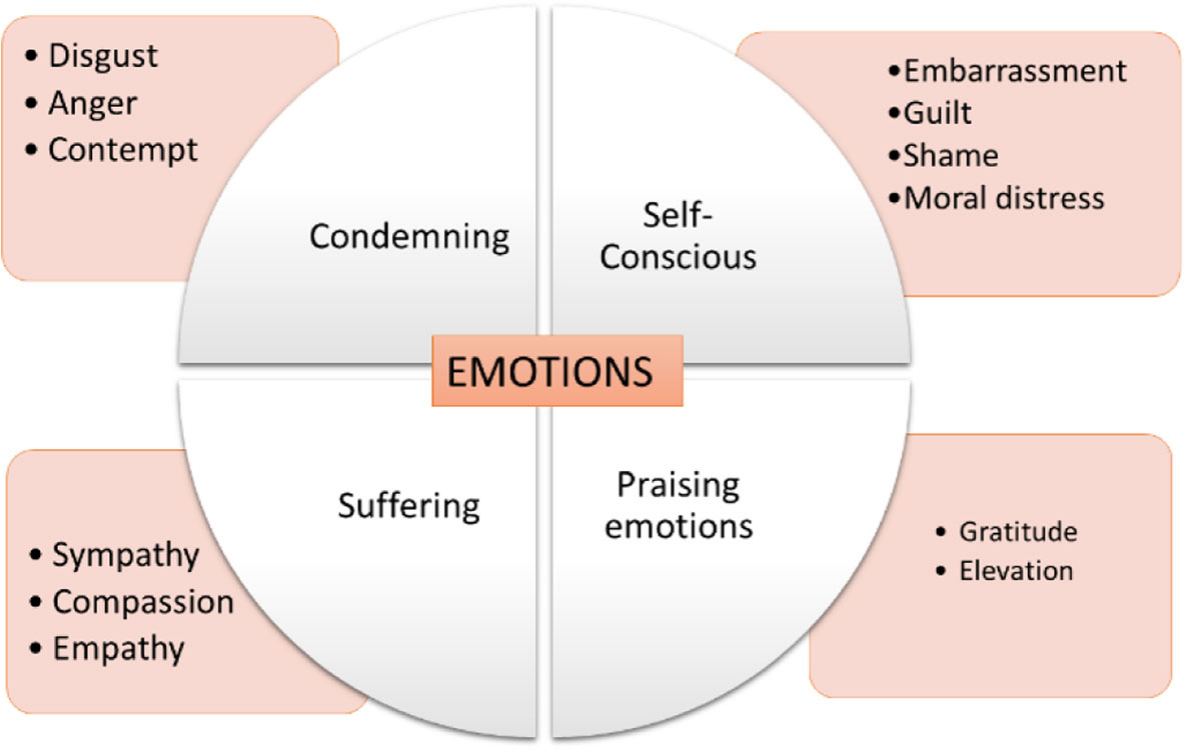
Moral emotions

We present one main category, “emotions and feelings in caring” relate with moral emotions. This category was strongly linked to the caring process. The subcategories were condemning emotions, self-conscious emotions, praising emotions and suffering emotions. Figure 1 shows the category with the different subcategories [20].

### Condemning emotions

These emotions are related to the negative feelings nurses experience when they have to take part in ethical situations related to the care given by other professionals. In this subcategory, we could include feelings such as disgust, anger or contempt, which sometimes arise in extreme situations when the treatment that is given is not appropriate or when practices could be described as inhuman or violent, e.g. compulsory. The nurses expressed these feelings from emotions when their viewpoint was not taken into account in the decision-making process. They were not allowed to be involved in the planning of good care and felt that the medical treatment displaces nursing care and does not include it as a part of the patient’s treatment process. When nurses talk about the treatment of pain, they see it as an ethical matter and, when it is not addressed adequately, this generates a feeling of anger in them.

“ … *that’s why I get annoyed. Because, despite having many tools, we still have to keep asking whether or not to give the patient a painkiller. This triggers one’s temper, to say: ‘come on, this person is suffering’, e.g. vascular patients who have had pain for many hours, it’s very easy to give them something.”* GF:R4 [6]

The nurses also felt anger in situations when the assessment did not involve both patient and family and when actions that were unnecessary for the patient were performed. The feeling was that some professionals only focus on an organ or a set of organs, while some view the person as a whole, taking account of other aspects that are an important part of life.

*"( … ) in my experience, the patient's life is prolonged as much as the doctor wants and maybe the patient has written not to resuscitate him or her in the event of cardiac arrest. I don't understand it, you are telling me there's nothing we can do and we are filling them up with tubes, serums, catheters, drugs... if there is no chance of waking up, why are you prolonging unnecessary agony? The patient can't feel a thing, but what about the family members?*

*“You should see how they suffer, how they cry... it tears your heart out. Even if you ask the doctor ‘what are you doing?’, if he thinks that they have to keep on, they keep on... Finally, the patient will die, but adorned like a Christmas tree, not as a human being. Yesterday, a woman died on my watch about whom, for more than a week, we had only heard “there's nothing we can do”, but she wasn't short of anything, noradrenaline, tubes, catheters, serums... There comes a time when you get tired of speaking and not being listened to.” GF: R10* [19]

### Self-conscious emotions

Self-conscious emotions provoke negative feelings like shame, guilt or embarrassment. Nurses report that, when they had experienced a situation in which they believed they could have helped the patient differently, but the conditions at the time did not permit it, they felt guilty about having taken part in the process. Nurses feel vulnerable in these situations where they cannot act.

The emergency nurses highlighted the fact that the lack of teamwork between professionals harms the patients and provokes these emotions. The lack of teamwork also impairs the individual effort and the relationship between all the participants in the health-care team.

*“(...) the professional relationships must be based on consensus within the team, because, if there is no dialogue, nothing works in the optimal way. At the hospital, the team doesn't talk, there is no dialogue, they don't work well together... this often results in many ‘loose ends’ and a lack of understanding about what happened... each member plays his part and it goes as it goes (...) this makes me feel ashamed about not being able to solve it (...).” ENF2* [2]

Nurses feel shame and guilt because they see clearly that there is no teamwork and this affects the caring process.

### Praising emotions

Within this subcategory, we include the feelings that could be defined as satisfactory and positive; they arise when nurses feel that care has met its objectives and that there has been an appropriate response to needs. Nurses highlight the fact that these positive feelings gratify and motivate them to continue advancing and developing a more complete and satisfying nursing practice for both patients and professionals.

*“(...) helping people makes me feel fulfilled, you are next to them in very serious and critical situations and we are behind the care given at these difficult moments. We help them with their problems; help them to keep on living. Sometimes we find that we are powerless because we can’t do anything to help them... that’s the two sides of the same coin (...).” Enf2* [7]

Another participant highlights the need to do the right things to experience this kind of feeling, because it produces a significant degree of personal satisfaction as a professional.

*“(...) sometimes the situations fluctuate tremendously, we can go from one extreme to the other: from maximum satisfaction to the utmost helplessness. I am determined never to fail, I must be one hundred per cent. Feelings like this help me to act. It requires an extra effort because time is precious and perhaps we have to resolve situations that may endanger a patient’s life. At the same time, they help me to develop as a human being ( … ).” Enf8* [4, 5]

When nurses participate in this decision-making process, they feel good in spite of the difficulties that may arise in the situations they must face.

*“(...) in emergencies, things happen quickly and we often don’t have time to stop and think, I feel that I am part of the situation I am in ( … ).” Enf8* [8]

### Suffering emotions

The nurses felt that care, as seen from a nursing perspective, has a sensitive dimension that goes beyond the patient’s own healing and, when this is in conflict, it dehumanises the assistance. Nurses believe that the caring perspective must consider a special moral sensitivity in order to respond to the needs of the patient.

The informants state that distress coming from a morally negative emotion is the main source of moral distress. Moral distress is made up of emotions that appear when, for various reasons, it is impossible to follow the right course.

Nurses suggest that there are external constraints that cause these situations, such as the institutional structure and its bureaucratisation, as well as the strict hierarchy that exists among professionals in hospital. This situation has its origins in a power structure, more or less open, and, in other cases, invisible influences in the nursing/caring process.

According to one of the nurses:

*“(...) no, you are not taken into account for anything. If you were, sometimes things would have gone differently, at least from my own experience. You can argue, discuss, share opinions, it’s all useless. According to them, they are the captain and a sailor has to obey. Sometimes you are certain that the patient is going to die, but we still purify the blood and give antibiotics. We treat them with the most advanced and expensive therapeutic facilities Do you have any idea how much a haemofilter costs? Do you know how much unnecessary spending is generated? Do you know how much suffering we cause people? It is hard to live with this, I get angry, we talk about it with our colleagues... you can't do anything and feel helpless. However, when I see these atrocities, I tell them: ‘don't ever do that to me’. The most distressing thing is when the patient's family comes in and you see that agony. It breaks my heart and I realise that I am part of this...” Enf10* [9]

The informants sometimes felt that they were used to reaffirm the treatment and they did not have enough power to be the patient’s advocate. The following informant tells us about her experiences.

*“(...) no, they don't ask you. They very seldom do, but, if they do, it’s because they are searching for reaffirmation of their opinion and to be told that they are doing the right thing.” Enf3* [8]

*“(...) No, no, we don’t take an active part. Everything is under their control, everything is medicalised. Until the day arrives when nurses are on the same level as doctors and their work is valued by the medics, it will be very difficult for nurses to take part in the decision-making process when confronted by ethical issues (...).” Enf 4* [9]

The nurses say that they want to participate in the processes, bringing their experience and knowledge, but they feel that their opinions not are taken into account.

*“(...) Nowadays, nurses are in the clinical sessions, but their opinion is not taken into account; this should change gradually, the nurse knows the patients and defends them from aggressions that might occur even from health professionals. They do not usually take account of the information we provide, Physicians make decisions one hundred per cent of the time based on subjective criteria, which appear to be the only valid ones (...).” Enf 6* [8]

## Discussion

The overall impression from the findings from this extensive material was that nurses were preoccupied with existential thoughts about positive and negative moral emotions derived from caring relationships, such as emotions.

Moral emotions are linked to welfare, to do good and avoid doing bad. The present results found that the nurses who participated in the study indicated aspects that confirmed the existence of moral emotions that influence the caring process, sometimes positively and sometimes negatively.

Nurses are likely to feel condemning emotions like anger when assessing a situation relating to the patient and his/her family which goes against their view of the way things should be done and when they believe that action that is unnecessary for the patient could be avoided.

To do good from a nursing perspective is to take account of dimensions including the relationship between the patient and family. This perspective often differs from other sciences which focus on the biomedical perspective [11].

From the perspective of condemning emotions, anger is linked to the interests of others rather than to themselves. From this perspective, anger is a motivational force that energises the individual to defend situations in order to provide better care and avoid damage to the patient [10].

We found that this type of negative feeling constantly recurred in the emergency practices and was a topic of consensus among the interviewed nurses. Nurses need to develop their role in the team and other professionals need to include them in the ethical decisions. Other studies have shown the need for a nursing perspective in similar situations [26, 27].

Emotional responses from nurses in these situations vary a great deal. The informants state that a morally negative emotion is the main source of moral distress. Moral distress appears when, for various reasons, it is impossible to follow best practice and is independent of context-given specific preconditions: when nurses are morally sensitive to the patients’ vulnerability, when nurses experience external factors preventing them from doing what is best for the patient and when nurses feel that they have no control over the specific situation [28].

This gives the professionals a sense of helplessness, frustration, anger, resignation and guilt. What is worse, it can provoke states of depression associated with the loss of professional integrity, feelings relate with self-consciousness moral feelings.

Nurses has suffering feelings from the most common sources are excessively aggressive treatments, the misuse of resources, a lack of communication between professionals and patients, treatment goals that are poorly defined and poorly understood by all the members of the care team, a lack of respect for the will of the patient and the loss of continuity of care due to a lack of collaboration and consensus; both excessive interventions and the therapeutic neglect of patients could result from the latter actions [20].

The nurses suggested that there are external constraints that cause these situations, such as the institutional structure and its bureaucratisation, as well as the strict hierarchy that exists among professionals in hospitals. Hierarchy often results in the abuse of power and this then results in internal conflicts, more or less open, and, in other cases, invisible [29].

The nurses feel that they are the patients’ advocates and they cannot simply be governed by feelings of resignation and pessimism. They need to do something more. Nurses need support, strategies and solutions from the organisation to demonstrate their role as the patients’ advocates [30]. The collaboration between nurses and doctors could lessen feelings of moral distress if they felt included in the decision-making process [31]. They need to participate in these interdisciplinary teams. However, the interviewed nurses felt that clinical practice was far removed from achieving an adequate minimum of inter-relationships and, according to them, this only exists in the theoretical discourse [32].

Positive emotions are also present in clinical practice, even if, in many cases, emergency situations can be dramatic. These situations can, for example, give the professionals emotions such as gratitude and satisfaction. These emotions arise when nurses see that care meets the predicted goals and they have been able to respond to the needs. Positive emotions are beneficial for the professional experience [33].

The nurses point out that these positive feelings gratify and motivate them to continue advancing and developing a successful practice for both patients and professionals. Positive feelings prevent emotional exhaustion and help to prevent bad confidence [34].

The expression of care arises from a unique situation involving the nurse and the patient where both have expectations of a result. It is a unique and specific situation that cannot be repeated. To understand these relationships, it is necessary to contextualise instead of generalising when it comes to worrying about the principles that guide the action. Caring professionals are concerned about the person they care for. This creates feelings that give meaning to the interviewed professionals in their daily practice. If the professionals are aware of positive and negative moral emotions, this will help them to reach levels of personal satisfaction, self-fulfilment and moral reinforcement [35].

From this respect, the moral duty of health professionals not only lies in the effective exercise of their profession, from a technical point of view, it is also ethical and aesthetic experience implies the creation and/or appreciation of caring situations.

The praising emotions are living like a positive feeling is reinforced when they are praised for their work and this leads to emotional well-being which improves their quality of life from both a personal and a professional point of view [36].

### Limitations

Qualitative studies do not attempt to generalize results and therefore have some limitations. The present study was limited to a small sample size, which is characteristic of qualitative methods. The purpose of using the content analysis process was to interpret experiences based on an in-depth analysis of single cases rather than to generalise across a large number of cases.

When performing a content analysis interpretation, we do not expect to find a single universal truth, but instead we search for possible meanings in a continuous process. There is always more than one way to analyse and interpret data and the results of this study represent one of several possibilities.

The present findings illustrate the experiences of nurses. This research was conducted only with female nurses and could be biased in its results, but this could be the basis for future interventional studies and further dialogue in the ethical setting in clinical practice including gender perspective.

## Conclusions

The “moral emotions” contain feelings, some negatives and other positives, like shame, guilt, sympathy, empathy, contempt, anger, disgust, moral distress, joy and happiness Moral emotions are connected to the caring process in emergency and critical situations and so, from a nursing perspective, the study of moral emotions brings into play a larger array of feelings that will help us to understand the dynamics of the relationships involving the patients, the families, other professionals or institutions. It is therefore necessary, in a critical and rational manner, to develop a multidimensional analysis of care including both anthropological and ethical aspects and as much in its technical aspects as in its anthropological and ethical aspects. It is crucial not to ignore these emotions, because they are present in all caring actions.

The engagement between nursing practice and patient in vulnerable situations such as emergencies has a strong emotional element. A patient may elicit compassion, concern, pity or indeed anger or frustration. The nurses felt that they were unable to develop caring science because technological tasks play a greater part than in the caring process in place of the human dimension of care.

The nurses felt negative moral emotions like anger and frustration when restrictions affected the human dimension of quality of care. This was a problem, because they were unable to see any possible way of developing as professionals, to create a new kind of human care where the technology is involved but is not the main objective.

When nurses feel that they are working from a compassionate care perspective, this generates positive feelings like sympathy or happiness both for the staff and for the patients and their families. These aspects are very important and are the main aim of the nurses’ work. A nurse’s knowledge and skill are important forces that can contribute to the power to influence patient care in an ethical manner. This power comes from the nurse’s knowledge and expert skill.

To influence patient care, a nurse needs to be aware and also needs to understand the influence of moral emotions. This knowledge arms the nurse with power in the decision-making process relating to patient care. A nurse who understands his/her moral emotions can use this understanding to influence the health-care team and can apply it to the caring process by influencing both actions and behaviour.

## Data Availability

The raw data supporting the findings presented in this study will be available from the corresponding author upon request.

## Abbreviations

ALS: Advanced life support
ED: Emergency department
FG: Focus group
EMS: Emergency medical system
